# Midterm Blood Pressure Variability Is Associated with Poststroke Cognitive Impairment: A Prospective Cohort Study

**DOI:** 10.3389/fneur.2017.00365

**Published:** 2017-07-28

**Authors:** Shan Geng, Na Liu, Pin Meng, Niu Ji, Yong’an Sun, Yingda Xu, Guanghui Zhang, Xiaobing He, Zenglin Cai, Bei Wang, Bei Xu, Zaipo Li, Xiaoqin Niu, Yongjin Zhang, Bingchao Xu, Xinyu Zhou, Mingli He

**Affiliations:** ^1^Department of Neurology, The Affiliated Lianyungang Hospital of Xuzhou Medical University, Lianyungang, China

**Keywords:** stroke, cognitive impairment, Montreal Cognitive Assessment, blood pressure variability, subfactors

## Abstract

**Objective:**

The aim of this study was to investigate the relationship between blood pressure variability (BPV) and poststroke cognitive impairment (PSCI).

**Methods:**

Seven-hundred ninety-six patients with acute ischemic stroke were included in this study. Midterm BPV was evaluated by calculating the SD and coefficient of variation (CV, 100 × SD/mean) of systolic blood pressure (SBP) and diastolic blood pressure during the 7 days after stroke onset. Cognitive function was assessed using the Montreal Cognitive Assessment (MoCA) at admission and at all follow-up visits. Patients with MoCA scores <26 were considered to have PSCI.

**Results:**

The incidence of PSCI reached its peak (72%) 3 months after stroke onset and decreased to 30.3% at 12 months poststroke. After adjusting for covariables, the increase in the prevalence of PSCI at 3 months was independently associated with increases in the CV of blood pressure during the 7 days after stroke [odds ratios and 95% CI for patients in the second to fifth quintiles of SBP CV were 2.28 (1.18, 4.39), 2.33 (1.18, 4.62), 2.69 (1.31, 5.53), and 4.76 (1.95, 11.67), respectively]. Sub-analysis of the MoCA scores revealed that the patients had impairments in visuoperceptual abilities and executive functions, as well as in naming and delayed recall (*p* < 0.05).

**Conclusion:**

Midterm BPV during the early phase of acute ischemic stroke is independently associated with PSCI, especially in the visuoperceptual, executive, and delayed recall domains.

**Clinical Trial Registration:**

http://www.chictr.org.cn, identifier ChiCTR-TRC-14004804.

## Introduction

Poststroke cognitive impairment (PSCI) involves deficits in memory, comprehension, perception, language, and executive function (1). In the past 10 years, PSCI has been the subject of much attention, as it leads to poor life quality, slow functional recovery, and higher mortality. Current evidence suggests that PSCI occurs is about two-thirds of patients during the acute phase of ischemic stroke (2), and that 20–30% of patients with PSCI will develop dementia (3).

Multiple factors have been found to lead to progression of cognitive impairment and dementia after stroke. These include older age, family history, genetic variations, poor educational status, vascular comorbidities, and depression (4). Recently, a cohort study of 6,506 elderly individuals who were followed up for 8 years indicated that an increase of 1 SD in the coefficient of variability of systolic blood pressure (SBP) or diastolic blood pressure (DBP) is associated with an increase of about 10% in the risk of dementia (5). Higher visit-to-visit blood pressure variability (BPV) has also been proved to be associated with cognitive function impairment in the elderly (6). BPV has also been shown to be associated with neurological impairment (7), progression of brain white matter lesions (8), hemorrhagic transformation after ischemic stroke (9), and poor long-term prognosis (10). However, it is still unclear whether higher BPV is a cause or consequence of PSCI.

Poststroke cognitive impairment is not a unitary disease, but involves deficits in multiple domains. However, prospective follow-up studies regarding long-term domain-specific cognitive impairments are rare (11). The aim of this study was to investigate the relationship between BPV and PSCI and the prevalence of domain-specific cognitive impairment.

## Materials and Methods

### Study Design and Participants

We carried out a single-center, prospective, observational study. The protocol of this study was registered with Chinese Clinical Trial Registry (ChiCTR-TRC-14004804; http://www.chictr.org.cn/index.aspx) and derived from the expanded content of “stroke risk assessment form” and “follow-up table” determined by “stroke risk factors screening and intervention project,” People’s Republic of China Health Committee. Acute stroke was defined as the sudden attack of neurological deficit of cerebrovascular cause for more than 24 h. Computed tomography (CT) and magnetic resonance imaging (MRI) were used to diagnose stroke, to distinguish hemorrhage and ischemia, and to divide up the infarcts into the anterior and posterior depending on the location and area. The infarct volume was calculated by the Pullicino formula (12). From January 2013 to June 2014, 986 consecutive patients with acute ischemic stroke were registered in the Stroke Registry database. The study was approved by the ethic committee of the First People’s Hospital of Lianyungang (No. Lianyungang-2012-06) and written informed consent was obtained from each patient or his/her proxy.

### Inclusion and Exclusion Criteria

Patients meeting all of the following criteria were eligible to participate in our study: acute ischemic stroke within 24 h of onset, identified signs of focal neurological dysfunction, the National Institutes of Health Stroke Scale (NIHSS) scores ≥4, and the total scores ≥2 of the fifth motor arm and the sixth motor leg on NIHSS, ages between 40 and 75 years and SBP ≥ 140 mmHg and/or DBP ≥ 90 mmHg.

Patients meeting any of the following criteria were excluded from the study: severe disturbance of consciousness identified by a level of consciousness score >1 on the NIHSS (21 patients); modified Rankin Scale score >1 before stroke onset (no patients); severe mental disorder or dementia (7 patients); serious systemic diseases or expected life span <90 days (24 patients); alanine transaminase or aspartate aminotransferase >2.0, which is at the upper limit of normal, or severe liver disease (23 patients); estimated glomerular filtration rate <30 ml/min/1.73 m^2^ or sever kidney disease (13 patients); aphasia (*n* = 36), inability to complete the Montreal Cognitive Assessment (MoCA) (*n* = 41); or being considered inappropriate (living or working non-local and have a lower degree of integrity) for the study (*n* = 25) (Figure S1 in Supplementary Material).

### Management and Measurement of Blood Pressure

Patients with acute ischemic stroke were treated according to the 2010 Chinese Guidelines for the diagnosis and treatment of acute ischemic stroke. The antihypertensive strategies were in accordance with the 2010 Chinese Guidelines for Prevention and Treatment of Hypertension (13, 14). During the first 24 h of hospitalization, SBP and DBP were controlled to between 140 and 159 mmHg, and 90 and 99 mmHg, respectively. Patients with SBP ≥ 180 mmHg and DBP ≥ 120 mmHg were given nitroglycerine or sodium nitroprusside intravenous pump. The rate of blood pressure reduction was controlled at ≤5 mmHg/h by adjusting the dose of the medication. For patients with SBPs of 160–180 mmHg and/or DBPs of 100–120 mmHg, single or combined antihypertensive drugs were administered. For patients with SBP < 160 mmHg and/or DBP < 100 mmHg at admission, no antihypertensive drugs were given. After the first 24 h of hospitalization, patients with SBP ≥ 140 mmHg and DBP ≥ 90 mmHg were given reasonable antihypertensive drugs.

Supine BP was measured by trained nurses using a standard mercury sphygmomanometer on the non-paralyzed arm on admission and every 4 h on days 1–7. In the stroke unit, BP was measured using a non-invasive BP monitoring system (Philips SureSigns VM6 monitor; Royal Dutch Philips Electronics Ltd., Amsterdam, Netherlands). Midterm BPV was evaluated by calculating the SD and coefficient of variation (CV, 100 × SD/mean) of SBP and DBP during the 7 days after stroke onset (15).

### Clinical Assessment

Ischemic stroke was defined as stroke identified by radiographic diagnosis (CT or MRI) and clinical diagnosis (16). The acute phase of ischemic stroke was defined as the 7 days following symptom onset (17). The infarct volume on CT/MRI (diffusion-weighted imaging/fluid attenuated inversion recovery) images was calculated using the Pullicino equation (net infarct volume = *L* × *W* × *H*/2; *L, W*, and *H* refer to the length, width, and height of the infarct lesion, respectively) (12). Thrombolytic therapy was defined as intravenous administration of recombinant tissue-type plasminogen activator (rt-PA) within 4.5 h of symptom onset or catheter-directed thrombolysis using rt-PA within 12 h of symptom onset.

### Judgment of Outcomes and Follow-up

All participants were followed up 14 days, 3 months, 6 months, and 12 months after onset by trained neurologists. In this study, cognitive function was assessed using the MoCA. Patients with MoCA scores ≤25 and education levels <12 years or those with MoCA scores ≤26 and education levels >12 years were considered cognitively impaired (18). A study by Nasreddine and his colleagues demonstrated that the MoCA has a sensitivity of 90% and a specificity of 87% when a cutoff score of 26 is used (19). It is also reported that the MoCA is an effective and brief tool for detecting cognitive impairment in the elderly (20) and is more in line with the criteria for mild cognitive impairment than the Mini-Mental State Examination in populations older than 60 years (21).

At the 1-year follow-up, 62 patients had died and 124 patients were lost (Figure S1 in Supplementary Material). The patient deaths were due to pulmonary infection, recurrence of cerebral infarction, cerebral hemorrhage, myocardial infarction, gastrointestinal bleeding, liver and kidney failure, or other complications. Loss to follow-up was due to the patients not agreeing to repeat the MoCA, or loss of contact due to changes in residence. Six-hundred ten patients were left at the 12-month follow-up.

### Sample Size Estimation

According to the sample size calculation formula for cohort studies:

$$n=\frac{{\left(u_{\alpha /2}\sqrt{2pq}+u_{\beta }\sqrt{p_{0}q_{0}+p_{1}q_{1}}\right)}^{2}}{{\left(p_{1}-p_{0}\right)}^{2}}$$

in which *p*_0_ is the estimated prevalence of cognitive impairment, *p*_1_ is the estimated prevalence of PSCI, *q*_0_ = 1 − *p*_0_, *q*_1_ = 1 − *p*_1_, *p* = (*p*_0 + _*p*_1_)/2, *q* = 1 − *p*. *u*_α_ and *u*_β_ are the significance testing statistic, α = 0.05, β = 0.10. The calculated sample size required for the study was 115, based on those estimations that the prevalence of cognitive impairment and PSCI were 12.7 and 30% (22, 23).

### Statistical Analysis

Normally distributed continuous variables are presented as means ± SDs and were compared using Student’s *t*-tests. Not normally distributed variables were presented as median (interquartile range) and were compared between groups using Mann–Whitney *U* test. Categorical variables are expressed as frequency and percentage and were compared using χ^2^ tests. To evaluate the association between midterm BPV and cognitive function, patients were divided into quintiles (Q1–Q5) according to the CVs of SBP and DBP during the 7 days after stroke onset. The odds ratios (ORs) and 95% confidence intervals (CIs) were calculated by logistic regression and the following confounders was adjusted age, gender, education degree (less than 12 years), hypertension, SBP and DBP on admission, CIV, location of infarction (cortex, cortex-subcortical, subcortical, brain stem, and cerebellum), HAMD, and thrombolytic therapy. *p* Values <0.05 were considered statistically significant. Data were analyzed using SPSS.v19.0.1 software package.

## Results

### Clinical Characteristics

The clinical characteristics of the 708 patients are presented according to the presence of cognitive impairment 3 months after stroke onset, the patients who had died and those lost to follow-up are excluded from the data (Table 1). The average age of these patients was 63.1 ± 10.0. Three-hundred eighty-three of the patients (54.1%) were men and 561 (79.2%) had less than 12 years of education. Compared to patients with no cognitive impairment, those with cognitive impairment were more likely to be older and have lower education levels and higher blood pressure on admission. They were also more likely to have a history of hypertension and lower NIHSS scores and to have received thrombolysis (Table 1). Patients with cognitive impairment also had higher CVs of blood pressure, cortical and subcortical infarction, large artery atherosclerosis, and small-artery occlusion, as classified by the guidelines of Trial of Org 10172 in Acute Stroke Treatment (TOAST) (Table S1 in Supplementary Material).

**Table 1:** Comparison of baseline characteristics between patients with and without cognitive impairment 3 months after onset.

Variable	Total (*n* = 708)	No cognitive impairment (*n* = 198)	Cognitive impairment (*n* = 510)	*t* value, *U* value χ^2^	*p*-Value
Age (mean ± SD, years)	63.1 ± 10.0	58 ± 11.6	65.1 ± 8.5	−8.972	<0.001
Males (*n*, %)	383 (54.1)	115 (58.1)	268 (52.6)	1.758	0.185
Body mass index (mean ± SD, kg/m^2^)	25.7 ± 2.8	25.6 ± 2.9	25.8 ± 2.8	−0.740	0.460
Less than 12 years of education (*n*, %)	561 (79.2)	115 (58.1)	446 (87.5)	74.783	<0.001
Hypertension (*n*, %)	624 (88.1)	160 (80.8)	464 (91.0)	14.114	<0.001
Hyperlipidemia (*n*, %)	397 (56.1)	112 (56.6)	285 (55.9)	0.027	0.869
Diabetes mellitus (*n*, %)	161 (22.7)	47 (23.7)	114 (22.4)	0.156	0.693
Coronary heart disease (*n*, %)	96 (13.6)	26 (13.1)	70 (13.7)	0.043	0.836
Atrial fibrillation (*n*, %)	139 (19.6)	46 (23.2)	93 (18.2)	2.257	0.133
History of TIA (*n*, %)	114 (16.1)	32 (16.2)	82 (16.1)	0.001	0.978
Current smoking (*n*, %)	211 (29.8)	63 (31.8)	148 (29.0)	0.534	0.465
Current drinking (*n*, %)	152 (21.5)	40 (20.2)	112 (22.0)	0.262	0.609
Systolic blood pressure (mean ± SD, mmHg)	168 ± 25.0	165 ± 24.8	169.1 ± 25.0	−2.002	0.046
Diastolic blood pressure (mean ± SD, mmHg)	107.9 ± 16.4	105.7 ± 16.5	108.7 ± 16.3	−2.148	0.032
Homocysteine (mean ± SD, μmol/l)	14.1 ± 1.7	14 ± 1.7	14.2 ± 1.7	−1.459	0.145
eGFR (mean ± SD, ml/min/1.73?m^2^)	95.6 ± 10.5	96.6 ± 10.6	95.2 ± 10.4	1.574	0.116
National Institutes of Health Stroke Scale on admission (median, interquartile range)	11.0 (4.0)	9.0 (3.0)	12.0 (4.0)	28,736.5	<0.001
Modified Rankin Scale on admission (median, interquartile range)	3.0 (0)	3.0 (0)	3.0 (0)	48,476.5	0.301
HAMD (mean ± SD, points)	3.5 ± 2.1	3.7 ± 2.1	3.4 ± 2.0	1.892	0.059
CIV (mean ± SD, cm^3^)	10.1 ± 2.1	10.1 ± 2.1	10.1 ± 2.1	0.273	0.785
Thrombolysis (*n*, %)	51 (7.2)	23 (11.6)	28 (5.5)	8.007	0.005

### MoCA Score and Incidence of PSCI

**Figure 1:**
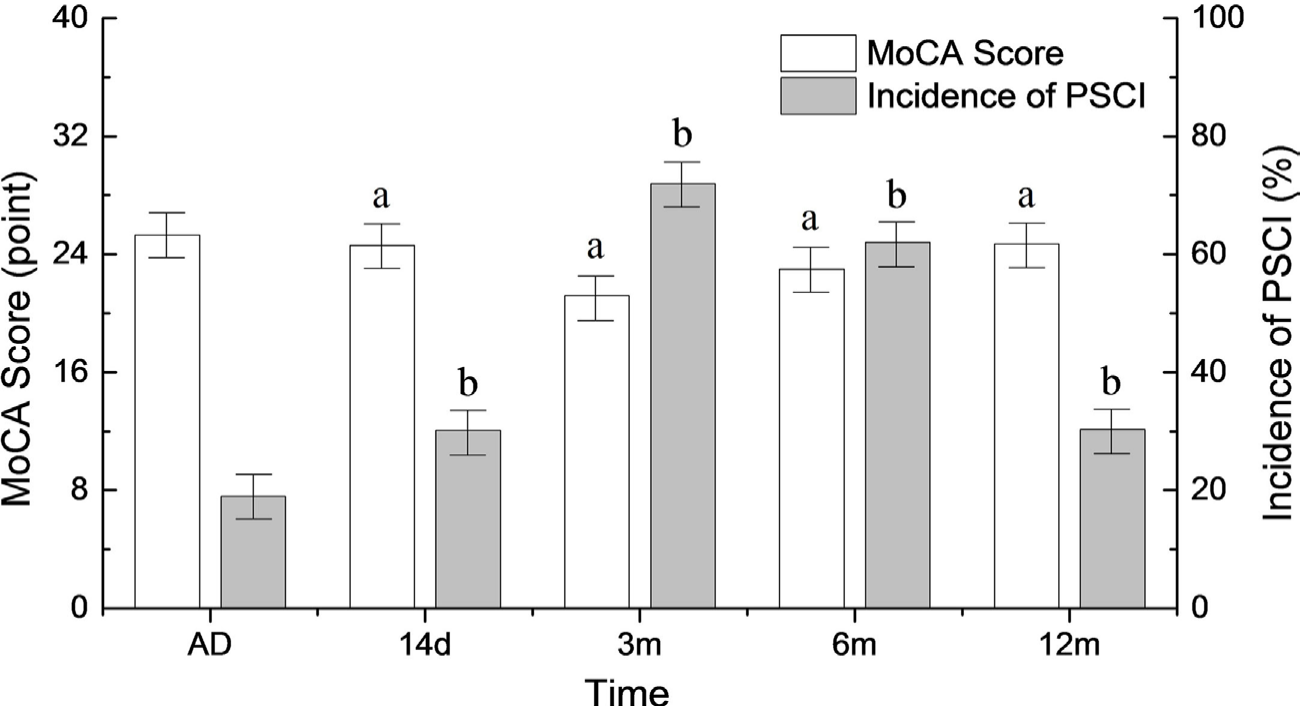
Changes in the incidence of poststroke cognitive impairment (PSCI) and MoCA scores during the follow-up period. AD, at admission. ^a^*p* < 0.05 compared to MoCA score at admission. ^b^*p* < 0.05 compared to PSCI at admission.

The incidence of PSCI began to rise at 14 days after onset (31.6%) and peaked 3 months after the stroke (72.6%). Six months after stroke onset, the incidence of PSCI began to decrease (66.5%). It reached the baseline level 12 months after the stroke (34.7%) (*p* < 0.05). A reciprocal trend for MoCA scores is shown in Figure 1.

### Cognitive Impairment and BPV

Simple comparisons between patients with no cognitive impairment and those with cognitive impairment indicated that the two groups had differences in age, education level (more vs. less than 12 years), hypertension, systolic and DBP at admission, TOAST classification, location of infarction, NIHSS score at admission, and the use of thrombolysis (all *p* < 0.05) (Table 1; Table S1 in Supplementary Material). Logistic analysis indicated that the CV of SBP had a significant association with PSCI, and that this relationship still existed after multi-parameter adjustments. When compared to patients in Q1, the adjusted ORs and 95% CIs of those in Q2-Q5 were 2.28 (1.18, 4.39), 2.33 (1.18, 4.62), 2.69 (1.31, 5.53), and 4.76 (1.95, 11.67) (Table 2). The risk factors for PSCI are shown in Table S2 in Supplementary Material. Men were more likely to have cognitive impairment than women (OR = 1.08, 95% CI = 1.06, 1.10), and lower education levels (less than 12 years) (OR = 7.72, 95% CI = 4.73, 12.61). These factors are both associated with an increased likelihood of cognitive impairment. Hypertension (OR = 2.07, 95% CI = 1.15, 3.72) and higher NIHSS scores at admission (OR = 1.33, 95% CI = 1.24, 1.44) were also more likely to increase the incidence of PSCI (Table S2 in Supplementary Material).

**Table 2:** Logistic regression analyses of CV during the 7 days following onset and cognitive impairment 3 months after onset.

Variables	E/R, *n* (%)	Unadjusted		Model I		Model II		Model III	
		OR (95% CI)	*p*-Value	OR (95% CI)	*p*-Value	OR (95% CI)	*p*-Value	OR (95% CI)	*p*-Value
**CV of systolic blood pressure**									
Q1 (4.5–7.7)	85/143 (59.4)	Ref	–	Ref	–	Ref	–	Ref	–
Q2 (7.8–8.1)	99/136 (72.8)	1.90 (1.11, 3.25)	0.019	1.95 (1.10, 3.43)	0.021	1.87 (1.01, 3.47)	0.047	2.28 (1.18, 4.39)	0.014
Q3 (8.2–8.5)	106/142 (74.6)	2.21 (1.26, 3.89)	0.006	2.23 (1.22, 4.08)	0.009	2.12 (1.11, 4.04)	0.022	2.33 (1.18, 4.62)	0.015
Q4 (8.6–9.2)	107/143 (74.8)	2.39 (1.33, 4.27)	0.003	2.40 (1.29, 4.48)	0.006	2.44 (1.24, 4.8)	0.010	2.69 (1.31, 5.53)	0.007
Q5 (9.3–15.1)	113/144 (78.5)	3.31 (1.61, 6.84)	0.001	3.29 (1.53, 7.08)	0.002	4.08 (1.75, 9.5)	0.001	4.76 (1.95, 11.67)	0.001
**CV of DBP**									
Q1 (3.7–6.9)	90/136 (66.2)	Ref	–	Ref	–	Ref	–	Ref	–
Q2 (7.0–7.5)	100/142 (70.4)	0.89 (0.51, 1.54)	0.675	0.83 (0.46, 1.49)	0.534	0.67 (0.35, 1.28)	0.225	0.57 (0.28, 1.15)	0.116
Q3 (7.5–8.2)	114/146 (78.1)	1.24 (0.69, 2.21)	0.474	1.14 (0.61, 2.10)	0.685	0.86 (0.43, 1.69)	0.655	0.98 (0.48, 2.01)	0.954
Q4 (8.2–8.8)	97/140 (69.3)	0.66 (0.36, 1.21)	0.178	0.60 (0.31, 1.13)	0.113	0.41 (0.2, 0.85)	0.017	0.31 (0.14, 0.68)	0.004
Q5 (8.8–14.6)	109/144 (75.7)	0.72 (0.35, 1.48)	0.369	0.63 (0.29, 1.36)	0.244	0.33 (0.13, 0.86)	0.024	0.27 (0.1, 0.74)	0.011

*Model I was adjusted for age and gender; Model II was based Model I plus education degree (less than 12 years), hypertension, SBP and DBP on admission, CIV and location of infarction (cortex, cortex–subcortical, subcortical, brain stem, and cerebellum); Model III was Model II plus National Institutes of Health Stroke Scale and thrombolytic therapy*.

*E/R, event/risk*.

### MoCA Score in Each Cognitive Domain 3 Months after Stroke Onset and CV during the 7 days after Stroke Onset

Table 3 shows the relationship between MoCA scores in each cognitive domain 3 months after stroke onset and the CV of SBP during the 7 days after stroke onset. Score in the visuoperceptual/executive and delayed recall domains were significantly different between patients with different SBPs. In addition, MoCA scores had an inverse relationship with the CV of SBP (all *p* < 0.05). Patients in Q4 and Q5 had lower scores in the naming domain than those in Q2 and Q3 (*p* < 0.05). Score on the abstraction domain were lower in patients in Q2 than in those in Q1 (*p* < 0.05). Patients in Q5 had lower scores in the orientation domain than those in Q1and Q2 (*p* < 0.05). Patients in Q4 had lower scores in the language domain than those in Q5 (*p* < 0.05). There were no significant differences in the cognitive domains of attention between the groups (all *p* > 0.05) (Table 3).

**Table 3:** The relationship of MoCA score in each cognitive domain at 3 months and CV of systolic blood pressure within 7 days of stroke onset.

	Q1 (*n* = 143)	Q2 (*n* = 136)	Q3 (*n* = 142)	Q4 (*n* = 143)	Q5 (*n* = 144)	*F* value	*p*-Value
Total scores	23.6 ± 3.4	22.1 ± 3.3	21.1 ± 3.6	20.3 ± 3.9	19.0 ± 3.9	32.874	<0.001
Visuoperceptual/executive	3.3 ± 1.3	2.7 ± 1.4	2.1 ± 1.5	1.5 ± 1.5	1.0 ± 1.3	64.339	<0.001
Naming	2.5 ± 0.7	2.4 ± 0.9	2.4 ± 1	2.6 ± 0.8	2.7 ± 0.6	3.333	0.010
Abstraction	1.8 ± 0.4	1.7 ± 0.6	1.7 ± 0.6	1.7 ± 0.5	1.8 ± 0.4	1.418	0.226
Orientation	5.1 ± 1.3	5 ± 1.3	5.2 ± 1.3	5.2 ± 1.1	5.4 ± 1.1	2.037	0.087
Attention	4.8 ± 1.5	4.7 ± 1.6	4.7 ± 1.6	4.7 ± 1.5	4.5 ± 1.6	0.461	0.764
Language	2.5 ± 0.8	2.5 ± 0.9	2.5 ± 0.9	2.6 ± 0.9	2.4 ± 0.8	1.211	0.305
Delayed recall	3.5 ± 1.6	3.1 ± 1.6	2.5 ± 1.6	1.9 ± 1.7	1.2 ± 1.7	43.874	<0.001

*Values are presented as mean ± SD*.

### Cognitive Impairment and Site of Cerebral Infarction

**Figure 2:**
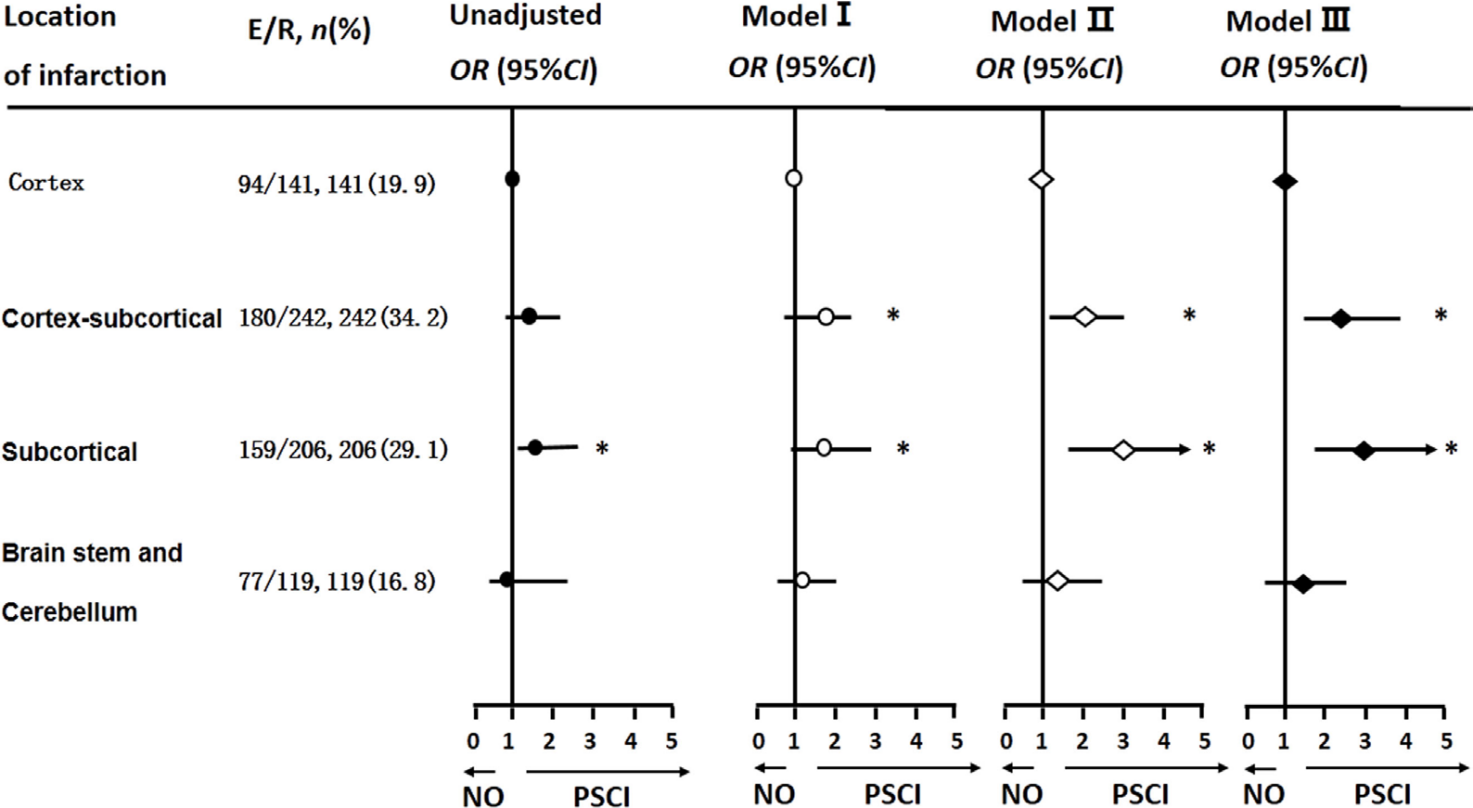
Logistic regression analyses of location of infarction and cognitive impairment 3 months after stroke onset. The multi-parameter model used is the same as that used in Table 2.

After adjusting for covariables, Cortical–subcortical and subcortical infarctions were more likely to lead to PSCI than cortical infarctions [ORs and 95% CIs were 2.03 (1.12, 3.65) and 2.86 (1.54, 5.33), respectively] (Figure 2).

## Discussion

Our findings suggest that high midterm BPV within 7 days of stroke onset is independently associated with an increased risk for PSCI 3 months after the stroke. Our findings also indicate that male sex, low education levels, high NIHSS scores, cortical–subcortical infarction, and subcortical infarction are risk factor for PSCI. In contrast, cardioembolism of TOAST classification has no effects on cognitive impairment after stroke.

Blood pressure variability is a complex phenomenon that includes short-term fluctuations occurring within a 24-h period, midterm fluctuations over different days, and blood pressure changes over weeks, months, and even years (24). These variations in BP are thought to be the result of complex interactions between extrinsic environmental and behavioral factors and intrinsic cardiovascular regulatory mechanisms (central neural, reflexive neural, and humoral influences) that are not yet completely understood (15). Most importantly, high short-term BPV is a predictor of target organ damage and future cardiovascular events (25, 26). High midterm SBP variability in daytime was a predictor of cardiovascular and all-cause mortality (27). In addition, a recent study has reported that higher midterm BPV was associated with progression of brain white matter lesions and lower cognitive function (28). The authors of the above study suggest that higher BPV is a risk factor for lower hippocampal volume and cerebral mircobleeds independent of average systolic and DBPs. In some analyses, therefore, the predictive value of BPV is even greater than that of average BP during treatment.

A recently study reported an increase in the incidence of stroke in China (29), which may result in an increased prevalence of PSCI. In our study, the incidence of PSCI reached its peak 3 months after ischemic stroke onset. Changes in the incidence of PSCI have an inverse relationship with MoCA scores. Consistent with our findings, the incidence of cognitive impairment 3 months after ischemic stroke was reported to be 71% in a study of 409 middle age and elderly patients with cerebral infarction (10). However, in the hospital-based cohort studies of Ihle-Hansen et al. (30) and Kandiah et al. (31), the incidence of PSCI was 37.5% 1 year after stroke and 37.32% 6 months after stroke. The broad range of reported PSCI incidence may be mainly attributed to differences in race, age, education level, location of stroke, and evaluation methods. We found that the prevalence of PSCI reaches its peak 3 months after stroke and decreases thereafter. It is, however, still unclear whether this trend is a consequence of transient cerebral function disorders and the recovery of stress injury.

The relationship between variability in blood pressure during the acute phase of ischemic stroke and lower cognitive performance 3 months after stroke onset was assessed using multiple parameter logistic regression analysis in our study. Independently of the confounding factors, SBP variability, but not DBP variability, was found to significantly predict the occurrence of cognitive decline. Consistent with our results, a recent epidemiological study (32) reported that day-to-day variability in SBP is significantly related to cognitive impairment (OR, 1.51; *p* = 0.02). In a study of 353 people, McDonald et al. (33) found that daytime variability in blood pressure is independently associated with greater decreases in total Cambridge Cognitive Examination and Mini-Mental State Examination scores after 5 years of fellow-up. Qin et al. (34) have suggested that higher visit-to-visit variability in systolic BP, but not mean systolic BP, is a predictor of cognitive decline. This stronger relationship can be explained by the fact that more information is provided by measuring the variability of BP than mean BP (35). In addition, the white-coat effect can be eliminated (36) and more target organ damage can be revealed than when using conventional BP measurements (37). However, Kilander et al. (38) suggested 24-h SBP was not associated with cognition. Other study of community-living adults also found no association between SBP and cognitive function (39). It may be explain from it, community-living adults were better able to automatically adjust the effect of systemic SBP on cerebral blood perfusion, which keep them with better baseline perfusion. Some studies have found higher DBP variability was related to poorer cognitive function (40), whereas in other studies no associated between variability in DBP and cognitive function was found (41). However, in our study, higher variability in DBP did not seem to be protective for cognitive decline.

Nevertheless, the potential mechanism for the relationship between high BPV and cognitive impairment is still unclear. Recently, a study demonstrated that higher variability in BP, especially in SBP, can predict the progress of arteriosclerosis (42), lead to subcortical lesions (43), and contribute to the pathogenesis of cerebral vascular disease (44). All of the above factors may have negative impacts on cognitive function. Most importantly, hypertension and further decreases in blood pressure may reduce the coupling efficiency of the neurovasculature and impair dynamic cerebral autoregulation, which would then result in cerebral hypoperfusion. This may then lead to neurological impairment (45) and lead to progressive neurodegeneration (46). Considering energy requirements and blood supply, result in a neuronal energy crisis and cerebral hypometabolism, which may in turn lead to Alzheimer’s disease-related pathology (47).

Sub-analysis of the MoCA scale scores indicated that scores in the visuoperceptual and executive functions domains, as well as those for delayed recall, were decreased in individuals with high BPV. This suggests that visuoperceptual abilities, executive function, and delayed recall are more vulnerable than other domains of cognition. Consistent with our findings, a recent study showed that basal ganglionic lacunar infarcts and cerebral small vessel injuries are related to dysfunctions in delayed memory (48, 49). In addition, periventricular white matter hyperintensities have significant associations with executive function deficits (50). Using the same cohort as that used here, we have previously reported that patients with large artery atherosclerosis based on TOAST classification have lower scores in the visuoperceptual ability and executive function, and delayed recall and attention domains after correction for covariables (51). The potential mechanisms underlying poststroke cognitive decline are still unclear, although the cerebral cortex and hippocampus are sensitive to cerebral ischemia and anoxia (52), which may be a potential mechanism for impairment of visuoperceptual abilities, executive function, and delayed recall after stroke.

We also observed that cortical–subcortical and subcortical infarctions are more likely to lead to PSCI. Our results are in agreement with those of Hilal et al., who, in a study of 550 patients, reported that subcortical gray matter atrophy, such as that indicated by lacunes and white matter lesions, is not only observed in dementia but is also found in the preclinical stages of cognitive impairment (53). Rocque et al. reported that patients with large carotid plaques are more likely to have cognitive impairment (54). Pathophysiologically, multi-vessel, extracranial atherosclerotic disease may cause chronic diffuse brain hypoperfusion, which may be associated with cognitive impairment (55).

Our study has some limitations. First, only ages between 40 and 75 years were included in our study and other inclusion and exclusion criteria operated will influence BP variability and could weaken the external validity of results. Second, the use of different antihypertensive agents and other medications may have affected the variability in blood pressure and the relationship between BPV and poststroke cognition. What’s more, the study also had selection bias, as only hospitalized patients were enrolled, although this was inevitable. Another limitation of this study is that obstructive sleep apnea, which is associated with both BPV and cognitive dysfunction, could not be measured.

## Conclusion

Higher midterm SBP variability during the acute stage of cerebral infarction was found to be associated prospectively and independently with increased risk of PSCI, especially in the domains of executive function, naming, and delayed recall. This highlights the need for neurologists to pay more attention to the variability of blood pressure in patients with acute ischemic stroke. Population-based prospective studies are required to confirm our conclusions.

## Ethics Statement

Study on optimizing control strategy of blood pressure for the prevention of stroke in rural community. Ethical Review and Approval Documents. Our hospital is to carry out “Study on optimizing control strategy of blood pressure for the prevention of stroke in rural community,” the ethics committee of our hospital has a review of relative medical ethics issue of the project.

## Author Contributions

Conceptualization: SG, NL, PM, NJ, and MH. Data curation: SG and MH. Formal analysis: SG and MH. Investigation: YS, YX, BX, ZL, XN, YZ, CX, and XZ. Methodology: SG, NL, PM, and MH. Project administration: SG, NL, PM, NJ, and MH. Resources: SG, NL, PM, NJ, YS, YX, GZ, XH, ZC, BW, BX, ZL, XN, YZ, CX, and XZ. Software: SG and NL. Supervision: SG and MH. Validation: GS, NL, PM, NJ, MH, YS, YX, GZ, XH, and ZC. Visualization: SG and NL. Writing—original draft: SG. Writing—review and editing: SG and MH.

## Conflict of Interest Statement

The authors declare that the research was conducted in the absence of any commercial or financial relationships that could be construed as a potential conflict of interest.

## Supporting Information

Shan Geng et al., study investigators. Midterm BPV Is Associated with poststroke cognitive impairment.

### Data Definition

Body mass index was estimated from the equation: height (m)^2^/weight (kg). Hypertension was defined as systolic blood pressure of ≥140 mmHg and/or diastolic blood pressure of ≥90 mmHg or taking antihypertensive drugs. Low density lipoprotein cholesterol of ≥4.14 mmol/l, and/or high-density lipoprotein cholesterol of <1.04 mmol/l, triglyceride of ≥2.26 mmol/l or taking lipid-lowering drugs were defined as dyslipidemia. Diabetes mellitus was defined as fasting plasma glucose of ≥7.0 mmol/l, HbA1c of ≥6.5%, or treatment with oral antidiabetic drugs or insulin. Diagnosis of atrial fibrillation was confirmed by electrocardiography. Current smoking was defined as smoking cigarettes continuously or accumulates for 6 months and smoking in 30 days before the survey. Current drinking was determined as drinking more than 20 g each time and more than once a week.

Figure S1 The flow chart of the study.

Figure S2 Curves of cumulative survival.

Table S1 Comparison of baseline characteristics of patients with and without cognitive impairment 3 months after onset.

Table S2 Logistic regression analyses of CV during the 7 days following onset and cognitive impairment 3 months after onset. Model I was adjusted for age and gender; Model II was based Model I plus education degree (less than 12 years), hypertension, systolic blood pressure and DBP on admission, CIV and location of infarction (cortex, cortex–subcortical, subcortical, brain stem, and cerebellum); Model III was Model II plus National Institutes of Health Stroke Scale and thrombolytic therapy. E/R, event/risk.

Table S3 Comparison of baseline characteristics of patients with and without cognitive impairment 12 months after onset.

Table S4 Logistic regression analyses of CV during the 7 days following onset and cognitive impairment 12 months after onset. Model I was adjusted for age and gender; Model II was based Model I plus education degree (less than 12 years), hypertension, systolic blood pressure and DBP on admission, CIV and location of infarction (cortex, cortex–subcortical, subcortical, brain stem, and cerebellum); Model III was Model II plus TOAST classification, TIA, NHISS, and modified Rankin Scale score. E/R, event/risk.

Table S5 The relationship of the loss at different time points and CV of BP within 7 days of stroke onset.

Datasheet S1 Continued Table 1.
