# Supplementary figures and images for: Midterm Blood Pressure Variability Is Associated with Poststroke Cognitive Impairment: A Prospective Cohort Study
Source: Front Neurol. 2017 Jul 28;8:365. doi: 10.3389/fneur.2017.00365 (PMC5532726; doi:10.3389/fneur.2017.00365)

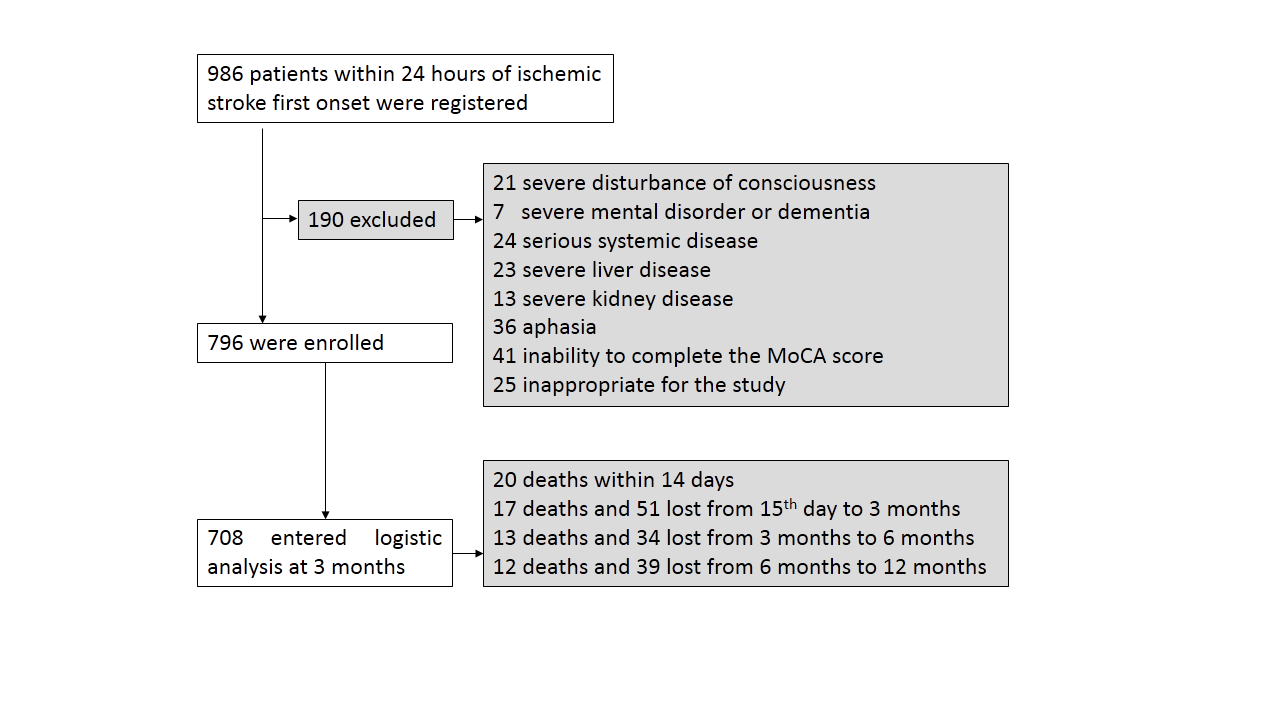

Supplement: Figure S1 — The flow chart of the study. [file image_1.tif]

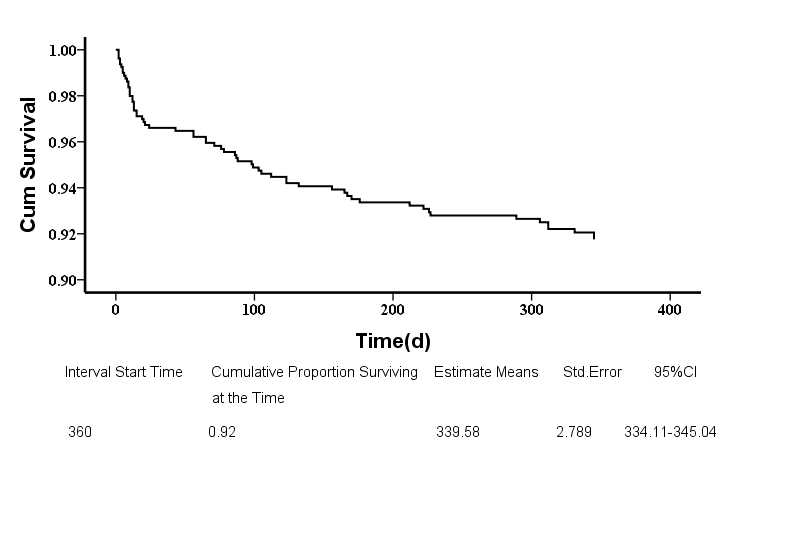

Supplement: Figure S2 — Curves of cumulative survival. [file image_2.tif]
