# Supplementary material for: Midterm Blood Pressure Variability Is Associated with Poststroke Cognitive Impairment: A Prospective Cohort Study
Source: Front Neurol. 2017 Jul 28;8:365. doi: 10.3389/fneur.2017.00365 (PMC5532726; doi:10.3389/fneur.2017.00365)
Supplement: Table S1 — Comparison of baseline characteristics of patients with and without cognitive impairment 3 months after onset. [file table_1.doc]

**S1 Table. Baseline characteristics of patient with and without cognitive impairment 3 months after onset.**

| Variables | Total  （n=708） | No cognitive impairment  （n=198） | Cognitive impairment  （n=510） | t value,U value  χ^2^ | P value |
| --- | --- | --- | --- | --- | --- |
| CV of SBP ( mean ± SD ) | 8.6±1.3 | 8.3±1.2 | 8.7±1.4 | -3.299 | 0.001 |
| Quintiles |  |  |  | 15.294 | 0.004 |
| Q1(4.5～7.7) | 143(20.2) | 58(29.3) | 85(16.7) |  |  |
| Q2(7.8～8.1) | 136(19.2) | 37(18.7) | 99(19.4) |  |  |
| Q3(8.2～8.5) | 142(20.1) | 36(18.2) | 106(20.8) |  |  |
| Q4(8.6～9.2) | 143(20.2) | 36(18.2) | 107(21.0) |  |  |
| Q5(9.3～15.1) | 144(20.3) | 31(15.7) | 113(22.2) |  |  |
| CV of DBP ( mean ± SD ) | 8±1.5 | 7.8±1.3 | 8±1.5 | -2.529 | 0.012 |
| Quintiles |  |  |  | 6.633 | 0.157 |
| Q1(3.7～6.9) | 136(19.2) | 46(23.2) | 90(17.6) |  |  |
| Q2( 7.0～7.5) | 142(20.1) | 42(21.2) | 100(19.6) |  |  |
| Q3(7.5～8.2) | 146(20.6) | 32(16.2) | 114(22.4) |  |  |
| Q4( 8.2～8.8) | 140(19.8) | 43(21.7) | 97(19.0) |  |  |
| Q5(8.8～14.6) | 144(20.3) | 35(17.7) | 109(21.4) |  |  |
| TOAST classification ( n, % ) |  |  |  | 19.082 | 0.001 |
| Large artery atherosclerosis | 110(15.5) | 21(10.6) | 89(17.5) |  |  |
| Small-artery occlusion | 212(29.9) | 48(24.2) | 164(32.2) |  |  |
| Cardioembolism | 28(4.0) | 9(4.6) | 19(3.7) |  |  |
| Other etiologyy | 103(14.6) | 44(22.2) | 59(11.6) |  |  |
| Undetermined etiology | 255(36.0) | 76(38.4) | 179(35.1) |  |  |
| Location of infarction ( n, % ) |  |  |  | 8.562 | 0.036 |
| Cortex | 141(19.9) | 47(23.7) | 94(18.4) |  |  |
| Cortex-subcortical | 242(34.2) | 62(31.3) | 180(35.3) |  |  |
| Subcortical | 206(29.1) | 47(23.7) | 159(31.2) |  |  |
| Brain stem and cerebellum | 119(16.8) | 42(21.2) | 77(15.1) |  |  |
| Family history ( n, % ) |  |  |  |  |  |
| Hypertension | 522(73.7) | 138(69.7) | 384(75.3) | 2.307 | 0.129 |
| diabetes | 170(24.0) | 43(21.7) | 127(24.9) | 0.793 | 0.373 |
| coronary heart disease | 96(13.6) | 26(13.1) | 70(13.7) | 0.043 | 0.836 |
| Stroke / TIA | 228(32.2) | 64(32.3) | 164(32.2) | 0.002 | 0.966 |
| Laboratory examination ( mean ± SD, mmol / L ) |  |  |  |  |  |
| Total cholesterol | 7.3±1.8 | 7.2±1.8 | 7.3±1.8 | -0.263 | 0.793 |
| Triglyceride | 2.3±0.3 | 2.4±0.3 | 2.3±0.3 | 0.813 | 0.417 |
| High density lipoprotein cholesterol | 0.9±0.1 | 0.9±0.1 | 0.9±0.1 | -1.305 | 0.193 |
| Low density lipoprotein cholesterol | 4.4±0.5 | 4.4±0.5 | 4.5±0.5 | -0.701 | 0.484 |
| Fasting blood glucose | 5.9±0.8 | 6±0.8 | 5.9±0.8 | 0.582 | 0.561 |
| Drugs after admission ( n, % ) |  |  |  |  |  |
| Lowering BP drugs | 601(84.9) | 171(86.4) | 430(84.3) | 0.467 | 0.494 |
| Antiplatelet drug | 662(93.5) | 186(93.9) | 476(93.3) | 0.086 | 0.769 |
| anticoagulant | 293(41.4) | 83(41.9) | 210(41.2) | 0.032 | 0.857 |
| statins | 613(86.6) | 167(84.3) | 446(87.5) | 1.186 | 0.276 |
| MoCA (Median, interquartile range ) | 26.0(1.0) | 26.0(1.0) | 26.0(1.0) | 47945.5 | 0.267 |
