# Supplementary material for: Midterm Blood Pressure Variability Is Associated with Poststroke Cognitive Impairment: A Prospective Cohort Study
Source: Front Neurol. 2017 Jul 28;8:365. doi: 10.3389/fneur.2017.00365 (PMC5532726; doi:10.3389/fneur.2017.00365)
Supplement: Table S2 — Logistic regression analyses of CV during the 7 days following onset and cognitive impairment 3 months after onset. Model I was adjusted for age and gender; Model II was based Model I plus education degree (less than 12 years), hypertension, systolic blood pressure and DBP on admission, CIV and location of infarction (cortex, cortex–subcortical, subcortical, brain stem, and cerebellum); Model III was Model II plus National Institutes of Health Stroke Scale and thrombolytic therapy. E/R, event/risk. [file table_2.doc]

**S2 Table. Single factor logistic regression analyses of CV within 7 days and cognitive function impairment 3 months after stroke onset.**

| Variables | Unadjusted | | Model Ⅰ | | Model Ⅱ | | Model Ⅲ | |
| --- | --- | --- | --- | --- | --- | --- | --- | --- |
|  | OR(95% CI) | P value | OR(95% CI) | P value | OR(95% CI) | P value | OR(95% CI) | P value |
| Age | — | — | 1.07(1.05,1.09) | 0.178 | 1.08(1.06,1.1) | <0.001 | 1.08(1.06,1.1) | <0.001 |
| Gender | — | — | 0.78(0.54,1.11) | <0.001 | 0.82(0.55,1.21) | 0.311 | 0.82(0.54,1.24) | 0.351 |
| Less than 12 years of education | — | — | — | 0.393 | 6.73(4.27,10.58) | <0.001 | 7.72(4.73,12.61) | <0.001 |
| Hypertension | — | — | — | — | 2.12(1.23,3.68) | 0.007 | 2.07(1.15,3.72) | 0.015 |
| SBP at admission | — | — | — | — | 1(0.98,1.02) | 0.896 | 1(0.98,1.03) | 0.795 |
| DBP at admission | — | — | — | — | 1.01(0.98,1.05) | 0.448 | 1.01(0.97,1.05) | 0.659 |
| Location of infarction | — | — | — | — | — | — | — | — |
| Cortex | — | — | — | — | ref | — | ref | — |
| Cortex-subcortical | — | — | — | — | 1.78(1.04,3.07) | 0.037 | 2.03(1.12,3.65) | 0.019 |
| Subcortical | — | — | — | — | 2.58(1.44,4.59) | 0.001 | 2.86(1.54,5.33) | 0.001 |
| Brain stem and cerebellum | — | — | — | — | 1.18(0.64,2.18) | 0.595 | 1.25(0.66,2.39) | 0.493 |
| NIHSS | — | — | — | — |  |  | 1.33(1.24,1.44) | <0.001 |
| Thrombolysis | — | — | — | — |  |  | 0.62(0.3,1.27) | 0.190 |
| CIV | — | — | — | — | 1.04(0.95,1.14) | 0.417 | 0.99(0.9,1.09) | 0.884 |

Model Ⅰ was adjusted for age and gender; Model Ⅱ was based Model Ⅰ plus education degree ( less than 12 years ), hypertension, SBP and DBP on admission, CIV and location of infarction ( cortex, cortex-subcortical, subcortical, brain stem and cerebellum ); ModelⅢ was Model Ⅱ plus NHISS and thrombolytic therapy. E /R : Event /Risk
