# Supplementary material for: Midterm Blood Pressure Variability Is Associated with Poststroke Cognitive Impairment: A Prospective Cohort Study
Source: Front Neurol. 2017 Jul 28;8:365. doi: 10.3389/fneur.2017.00365 (PMC5532726; doi:10.3389/fneur.2017.00365)
Supplement: Table S3 — Comparison of baseline characteristics of patients with and without cognitive impairment 12 months after onset. [file table_3.doc]

**S3 Table. Comparison of baseline characteristics of patient with and without cognitive impairment 12 months after onset.**

| Variable | Total  （n=610） | No cognitive impairment  （n=281） | Cognitive impairment  （n=329） | t value,U value  χ^2^ | P value |
| --- | --- | --- | --- | --- | --- |
| Age ( mean ± SD, yr ) | 63±9.9 | 62.8±10.3 | 63.4±9.1 | 0.532 | 0.466 |
| Males ( n, % ) | 329(53.9) | 195(69.4) | 230(69.9) | 0.019 | 0.891 |
| BMI ( mean ± SD,  kg / m^2^ ) | 25.8±2.8 | 25.8±2.9 | 25.8±2.7 | 0.000 | 0.999 |
| Less than 12 years of education ( n, % ) | 478(78.4) | 104(78.8) | 321(67.2) | 6.625 | 0.010 |
| Hypertension ( n, % ) | 537(88) | 58(79.5) | 367(68.3) | 3.754 | 0.053 |
| Hyperlipidemia ( n, % ) | 343(56.2) | 183(68.5) | 242(70.6) | 0.288 | 0.591 |
| Diabetes mellitus ( n, % ) | 137(22.5) | 332(70.2) | 93(67.9) | 0.268 | 0.605 |
| Coronary heart disease ( n, % ) | 81(13.3) | 370(69.9) | 55(67.9) | 0.139 | 0.710 |
| Atrial fibrillation ( n, % ) | 124(20.3) | 339(69.8) | 86(69.4) | 0.007 | 0.931 |
| History of TIA ( n, % ) | 105(17.2) | 342(67.7) | 83(79) | 5.276 | 0.022 |
| Current smoking ( n, % ) | 183(30) | 297(69.6) | 128(69.9) | 0.009 | 0.923 |
| Current drinking ( n, %) | 132(21.6) | 331(69.2) | 94(71.2) | 0.189 | 0.664 |
| Systolic blood pressure ( mean ± SD, mmHg ) | 167.9±25.3 | 166.9±25.2 | 170.3±25.4 | 2.336 | 0.127 |
| Diastolic blood pressure ( mean ± SD, mmHg ) | 107.8±16.6 | 107±16.7 | 109.7±16.4 | 3.490 | 0.062 |
| Homocysteine ( mean ± SD, μmol/L ) | 14.2±1.7 | 14.2±1.7 | 14.1±1.8 | 1.066 | 0.302 |
| eGFR, ( mean ± SD, ml/min/1.73m^2^ ) | 95.5±10.5 | 95.2±10.7 | 96.4±10.1 | 1.786 | 0.182 |
| NIHSS on admission (Median, interquartile range ) | 11.0(4.0) | 11.0(4.0) | 11.0(4.0) | 33364.5 | 0.003 |
| mRS on admission (Median, interquartile range ) | 3.0(0) | 3.0(0) | 3.0(1.0) | 35291 | 0.012 |
| HAMD ( mean ± SD, points ) | 3.5±2.1 | 3.5±2.1 | 3.4±2.1 | 0.187 | 0.666 |
| CIV ( mean ± SD, cm^3^ ) | 10.1±2.1 | 10±2.2 | 10.3±1.9 | 2.962 | 0.086 |
| thrombolysis ( n, % ) | 49(8) | 385(68.6) | 40(81.6) | 3.607 | 0.058 |

| CV of SBP ( mean ± SD ) | 8.6±1.3 | 8.5±1.3 | 8.7±1.5 | 4.809 | 0.029 |
| --- | --- | --- | --- | --- | --- |
| Quintiles |  |  |  | 5.579 | 0.233 |
| Q1(4.5～7.7) | 124(20.3) | 94(75.8) | 30(24.2) |  |  |
| Q2(7.8～8.1) | 109(17.9) | 78(71.6) | 31(28.4) |  |  |
| Q3(8.2～8.5) | 129(21.1) | 92(71.3) | 37(28.7) |  |  |
| Q4(8.6～9.2) | 126(20.7) | 84(66.7) | 42(33.3) |  |  |
| Q5(9.3～15.1) | 122(20) | 77(63.1) | 45(36.9) |  |  |
| CV of DBP ( mean ± SD ) | 8±1.5 | 7.9±1.4 | 8.2±1.6 | 4.662 | 0.031 |
| Quintiles |  |  |  | 4.224 | 0.377 |
| Q1(3.7～6.9) | 120(19.7) | 89(74.2) | 31(25.8) |  |  |
| Q2( 7.0～7.5) | 125(20.5) | 92(73.6) | 33(26.4) |  |  |
| Q3(7.5～8.2) | 129(21.1) | 89(69) | 40(31) |  |  |
| Q4( 8.2～8.8) | 111(18.2) | 75(67.6) | 36(32.4) |  |  |
| Q5(8.8～14.6) | 125(20.5) | 80(64) | 45(36) |  |  |
| TOAST classification ( n, % ) |  |  |  | 16.548 | 0.002 |
| Large artery atherosclerosis | 94(15.4) | 63(67) | 31(33) |  |  |
| Small-artery occlusion | 188(30.8) | 115(61.2) | 73(38.8) |  |  |
| Cardioembolism | 22(3.6) | 12(54.5) | 10(45.5) |  |  |
| Other etiologyy | 86(14.1) | 67(77.9) | 19(22.1) |  |  |
| Undetermined etiology | 220(36.1) | 168(76.4) | 52(23.6) |  |  |
| Location of infarction ( n, % ) |  |  |  | 3.605 | 0.307 |
| Cortex | 112(18.4) | 82(73.2) | 30(26.8) |  |  |
| Cortex-subcortical | 210(34.4) | 139(66.2) | 71(33.8) |  |  |
| Subcortical | 184(30.2) | 135(73.4) | 49(26.6) |  |  |
| Brain stem and cerebellum | 104(17) | 69(66.3) | 35(33.7) |  |  |
| Family history ( n, % ) |  |  |  |  |  |
| Hypertension | 446(73.1) | 113(68.9) | 312(70) | 0.063 | 0.802 |
| diabetes | 150(24.6) | 326(70.9) | 99(66) | 1.269 | 0.26 |
| coronary heart disease | 81(13.3) | 370(69.9) | 55(67.9) | 0.139 | 0.710 |
| Stroke / TIA | 198(32.5) | 285(69.2) | 140(70.7) | 0.149 | 0.700 |
| Laboratory examination ( mean ± SD, mmol / L ) |  |  |  |  |  |
| Total cholesterol | 7.2±1.7 | 7.2±1.8 | 7.2±1.7 | 0.002 | 0.967 |
| Triglyceride | 2.4±0.3 | 2.4±0.3 | 2.4±0.3 | 0.002 | 0.961 |
| High density lipoprotein cholesterol | 0.9±0.1 | 0.9±0.1 | 0.9±0.1 | 0.004 | 0.947 |
| Low density lipoprotein cholesterol | 4.4±0.6 | 4.4±0.6 | 4.5±0.6 | 1.132 | 0.288 |
| Fasting blood glucose | 5.9±0.8 | 6±0.8 | 5.9±0.8 | 0.329 | 0.566 |
| Drugs after admission ( n, % ) |  |  |  |  |  |
| Lowering BP drugs | 516(84.6) | 68(72.3) | 357(69.2) | 0.374 | 0.541 |
| Antiplatelet drug | 573(93.9) | 28(75.7) | 397(69.3) | 0.672 | 0.412 |
| anticoagulant | 255(41.8) | 244(68.7) | 181(71) | 0.355 | 0.551 |
| statins | 527(86.4) | 62(74.7) | 363(68.9) | 1.149 | 0.284 |
| MoCA (Median, interquartile range ) | 26.0(1.0) | 26.0(1.0) | 26.0(1.0) | 38117.5 | 0.525 |
