# Supplementary material for: Midterm Blood Pressure Variability Is Associated with Poststroke Cognitive Impairment: A Prospective Cohort Study
Source: Front Neurol. 2017 Jul 28;8:365. doi: 10.3389/fneur.2017.00365 (PMC5532726; doi:10.3389/fneur.2017.00365)
Supplement: Table S4 — Logistic regression analyses of CV during the 7 days following onset and cognitive impairment 12 months after onset. Model I was adjusted for age and gender; Model II was based Model I plus education degree (less than 12 years), hypertension, systolic blood pressure and DBP on admission, CIV and location of infarction (cortex, cortex–subcortical, subcortical, brain stem, and cerebellum); Model III was Model II plus TOAST classification, TIA, NHISS, and modified Rankin Scale score. E/R, event/risk. [file table_4.doc]

**S4 Table. Logistic regression analyses of CV within 7 days of onset and cognitive function impairment 12 months after onset.**

| Variables | E / R, n ( % ) | Unadjusted | | Model Ⅰ | | Model Ⅱ | | Model Ⅲ | |
| --- | --- | --- | --- | --- | --- | --- | --- | --- | --- |
|  |  | OR ( 95% CI ) | P value | OR ( 95% CI ) | P value | OR ( 95% CI ) | P value | OR ( 95% CI ) | P value |
| CV of SBP |  |  |  |  |  |  |  |  |  |
| Q1 (4.5～7.7) | 30/124(24) | ref | — | ref | — | ref | - | ref | - |
| Q2(7.8～8.1) | 31/109(28.4) | 1.21(0.65,2.23) | 0.551 | 1.2(0.65,2.23) | 0.554 | 1.17(0.62,2.19) | 0.630 | 1.22(0.64,2.33) | 0.547 |
| Q3(8.2～8.5) | 37/129(28.7) | 1.19(0.64,2.23) | 0.578 | 1.19(0.64,2.22) | 0.589 | 1.15(0.61,2.18) | 0.671 | 1.13(0.59,2.18) | 0.709 |
| Q4(8.6～9.2) | 42/126(33.3) | 1.47(0.78,2.78) | 0.235 | 1.46(0.77,2.76) | 0.242 | 1.45(0.76,2.79) | 0.260 | 1.62(0.83,3.19) | 0.160 |
| Q5(9.3～15.1) | 45/122(36.9) | 1.62(0.75,3.5) | 0.218 | 1.61(0.75,3.48) | 0.225 | 1.64(0.74,3.61) | 0.221 | 1.7(0.75,3.84) | 0.206 |
| CV of DBP |  |  |  |  |  |  |  |  |  |
| Q1(3.7～6.9) | 31/120(25.8) | ref | — | ref | — | ref | - | ref | - |
| Q2( 7.0～7.5) | 33/125(26.4) | 0.93(0.51,1.7) | 0.810 | 0.92(0.5,1.7) | 0.798 | 0.9(0.48,1.68) | 0.742 | 0.96(0.5,1.83) | 0.902 |
| Q3(7.5～8.2) | 40/129(31) | 1.13(0.62,2.08) | 0.688 | 1.12(0.61,2.06) | 0.708 | 0.99(0.52,1.9) | 0.986 | 1.12(0.57,2.2) | 0.739 |
| Q4( 8.2～8.8) | 36/111(32.4) | 1.12(0.58,2.17) | 0.738 | 1.11(0.57,2.16) | 0.749 | 1.03(0.51,2.09) | 0.938 | 1.08(0.52,2.26) | 0.828 |
| Q5(8.8～14.6) | 45/125(36) | 1.15(0.54,2.46) | 0.721 | 1.14(0.53,2.45) | 0.733 | 1.01(0.42,2.41) | 0.981 | 1.16(0.46,2.87) | 0.756 |

Model Ⅰ was adjusted for age and gender; Model Ⅱ was based Model Ⅰ plus education degree ( less than 12 years ), hypertension, SBP and DBP on admission, CIV and location of infarction ( cortex, cortex-subcortical, subcortical, brain stem and cerebellum ); ModelⅢ was Model Ⅱ plus TOAST classification, TIA, NHISS and mRS score . E /R : Event /Risk
