# Supplementary material for: Midterm Blood Pressure Variability Is Associated with Poststroke Cognitive Impairment: A Prospective Cohort Study
Source: Front Neurol. 2017 Jul 28;8:365. doi: 10.3389/fneur.2017.00365 (PMC5532726; doi:10.3389/fneur.2017.00365)
Supplement: Table S5 — The relationship of the loss at different time points and CV of BP within 7 days of stroke onset. [file table_5.doc]

**S5 Table. The relationship of the lose at different time points and CV of BP within 7 days of stroke onset.**

|  | Q1 | Q2 | Q3 | Q4 | Q5 | P value |
| --- | --- | --- | --- | --- | --- | --- |
| SBPV |  |  |  |  |  |  |
| 3 month | 10/158,6.3 | 10/157,6.4 | 13/157,8.3 | 11/157,7 | 7/157,4.5 | 0.740 |
| 6 month | 20/158,12.7 | 20/157,12.7 | 17/157,10.8 | 14/157,8.9 | 14/157,8.9 | 0.675 |
| 12 month | 28/158,17.7 | 28/157,17.8 | 24/157,15.3 | 22/157,14 | 22/157,14 | 0.788 |
| DBPV |  |  |  |  |  |  |
| 3 month | 12/158,7.6 | 11/157,7 | 6/157,3.8 | 13/157,8.3 | 9/157,5.7 | 0.524 |
| 6 month | 21/158,13.3 | 16/157,10.2 | 10/157,6.4 | 21/157,13.4 | 17/157,10.8 | 0.253 |
| 12 month | 25/158,15.8 | 23/157,14.6 | 18/157,11.5 | 35/157,22.3 | 23/157,14.6 | 0.111 |
